# Evidence for a sustained cerebrovascular response following motor practice

**DOI:** 10.1162/imag_a_00282

**Published:** 2024-08-29

**Authors:** Eleonora Patitucci, Davide Di Censo, Antonio M. Chiarelli, Michael Germuska, Valentina Tomassini, Richard G. Wise

**Affiliations:** Cardiff University Brain Research Imaging Centre (CUBRIC), Department of Psychology, Cardiff University, Cardiff, United Kingdom; Institute for Advanced Biomedical Technologies, University G. D’Annunzio of Chieti-Pescara, Chieti, Italy; Department of Neurosciences, Imaging and Clinical Sciences, University G. D’Annunzio of Chieti-Pescara, Chieti, Italy; Cardiff University Brain Research Imaging Centre (CUBRIC), Department of Physics and Astronomy, Cardiff University, Cardiff, United Kingdom; Institute of Psychological Medicine and Clinical Neurosciences, Cardiff University, Cardiff, United Kingdom; Helen Durham Centre for Neuroinflammation, University Hospital of Wales, Cardiff, United Kingdom; MS Centre, Department of Clinical Neurology, SS. Annunziata University Hospital, Chieti, Italy

**Keywords:** CBF, resting state, neuroplasticity, motor task

## Abstract

Motor tasks have been extensively used to probe neuroplasticity and the changes in MRI signals are often associated with changes in performance. Changes in performance have been linked to alterations in resting-state fluctuations of BOLD signal after the end of the task. We hypothesize that motor learning will induce localized changes in cerebral blood flow (CBF) sustained even after the execution of a motor learning task. We implemented a new motor task to probe neuroplasticity and mapped the associated cerebrovascular responses. Twenty healthy volunteers underwent two MRI sessions 1-week apart: a task session with a sequence learning task performed with a data glove and a control session. During each session, CBF and BOLD signals were acquired during the task and during two periods of rest, each lasting 8 min, before and after execution of the task. Evoked BOLD and CBF responses to the motor task were seen to decrease in a regionally specific manner as the task proceeded and performance accuracy improved. We observed a localized increase in resting CBF in the right extra-striate visual area that was sustained during the 8-min rest period after the completion of the motor learning task. CBF increase in the area was accompanied by a regional increase in local BOLD signal synchronization. Our observation suggests an important connection between neuroplastic changes induced by learning and sustained perfusion in the apparently resting brain followed task completion.

## 1 Introduction

The capacity of the human brain for functional reorganization throughout life, termed neuroplasticity, is now well recognized. Non-invasive neuroimaging enables investigation of the living human brain while learning new skills. In particular, motor learning tasks have been used to probe systems level neuroplasticity. The processes underlying neuroplasticity are thought to occur over different timescales. Microscale functional changes at the synaptic level such as long-term changes in neurotransmitter release, induction of long-term potentiation (LTP) leading to weakening and strengthening of existing synapses occur rapidly, whereas structural changes such as formation of new cellular components take place over a long period of time (Bruel-Jungerman et al., 2007;Butz et al., 2009;Holtmaat & Svoboda, 2009;Theodosis et al., 2008).

Changes over differing timescales have also been observed using MRI, where short-term functional changes have been reported during the execution of a motor task (Pascual et al., 1995;Shadmehr & Holcomb, 1997) and after its completion (Hardwick et al., 2013;Ungerleider et al., 2002), whereas evidence of structural changes has been documented over weeks or months (Chapman et al., 2015;Scholz et al., 2009). Functional magnetic resonance imaging is particularly adapted to investigating changes of brain activity over the timescale of seconds to minutes. This has led to the use of task-based fMRI to investigate neuroplasticity either dependent on experience gained with the task performed during the fMRI session or on training carried out between fMRI scan sessions (Tamás Kincses et al., 2008;Thomas et al., 2009). Furthermore, the observed functional changes in BOLD signal are often associated with improved performance (Doyon & Benali, 2005;Floyer-Lea & Matthews, 2004;Jueptner et al., 1997;Toni et al., 1998).

In contrast to research studies focusing on brain activity changes during a task, less is known about the existence and potential role of sustained changes in brain activity in the minutes following the completion of a task, such as motor training. The study of resting-state networks (RSN) has revealed alterations in functional connectivity (FC) following learning, suggestive of altered patterns of brain activity after a task. The neuronal circuitry underlying the observation of altered RSNs is thought to play an important role in supporting the execution of behaviour (Miall & Robertson, 2006). The association between altered resting activity and changes observed during task performance has been studied (Xiong et al., 2009). The strength of correlation within and between networks has behavioural relevance (Guerra-Carrillo et al., 2014), and given that experience-dependent sensorimotor plasticity can induce changes in BOLD resting-state (RS) fluctuations (Amad et al., 2017;Ge et al., 2015;Taubert et al., 2011;H. Zhang et al., 2014), fMRI has been proposed as an effective measure of plasticity. Thus, the study of RS is particularly adapted to highlight neuroplastic modification (Buckner & Vincent, 2007;Guerra-Carrillo et al., 2014).

BOLD-RS can be used to investigate long-distance FC and connectivity changes have been reported following motor training (Albert, Robertson, Miall, et al., 2009) and they have been associated with performance improvement (Vahdat et al., 2011), indicating that BOLD-RS may be used to probe the networks underlying the consolidation of new motor skills (Sami et al., 2014), yet there exist a notable gap in comprehending the impact of training on short-distance (local) FC. Local FC can be investigated using regional homogeneity (ReHo) analysis (Zang et al., 2004). ReHo evaluates the similarity of BOLD signal within specific brain regions, and it is thought to reflect diverse neurophysiological alterations (L. Deng et al., 2016;Jiang & Zuo, 2016). Recent findings indicate a robust correlation between ReHo and glucose metabolism (S. Deng et al., 2022). Consequently, ReHo has been employed to investigate local changes associated with rehabilitation after stroke (Yin et al., 2013), expertise acquisition (Dong et al., 2014;K. Zhang et al., 2021), and 2 weeks of motor training (Liu et al., 2022), positioning ReHo as a promising methodology for investigating local plasticity.

It is noteworthy that RS fMRI relies on temporal fluctuations of the BOLD signal over the timescale of a few seconds to a few tens of seconds. It does not reveal changes in neuronal, vascular, or metabolic activity over longer timescales. Furthermore, inference of altered neuronal activity from BOLD signal is complicated by the mixed nature of the physiological changes that underlie the BOLD response including variation of cerebral blood flow (CBF), cerebral metabolic rate of oxygen (CMRO_2_), and venous blood volume (Blockley et al., 2013). However, arterial spin labelling (ASL) MRI may be used to map CBF changes sustained over longer timescales (Mozolic et al., 2010), with the advantage that CBF is a quantitative physiological parameter more closely linked to neuronal activity and more directly reflective of vascular function than the BOLD signal.

Under the assumption of healthy neurovascular coupling (Iadecola, 2017), we expect CBF to reflect changes not only in underlying neuronal activity over short timescales seen for stimulus responses and spontaneous fluctuations in neuronal activity, but also for long-term changes in neuronal activity associated with brain plasticity. Additionally, we may expect sustained changes in metabolic activity or cerebrovascular function that support longer term alteration and reorganization of neuronal activity. Following from the observation that altered resting-state fluctuations following motor learning are evidence of changes in neuronal behaviour even during apparent rest after the completion of a task (Guerra-Carrillo et al., 2014), we hypothesize that there are observable sustained increases in CBF in motor-relevant brain regions following the execution of a motor learning task.

To investigate this hypothesis, we implement a new motor task, conducted over a period of 12 min, to probe neuroplasticity and use it to study the voxel-wise changes in resting CBF over a period of 8 min following the task. A BOLD-ReHo analysis (Zang et al., 2004) was performed to investigate changes in local synchronization of BOLD signal after the task. Furthermore, an FC analysis was also conducted to investigate whether, after completion of the task, there are changes in long-range BOLD FC.

## 2 Materials and Methods

### 2.1 Participants

Twenty healthy volunteers (age: 27.5 ± 3.8 years; 11F/9M) underwent two MRI sessions 1-week apart. Before each session, participants gave written informed consent and were screened for MRI compatibility.

Individuals with neurological or psychiatric disorders, as well as those taking prescribed medication, were excluded from the study participation. Participants were instructed to maintain their usual intake of food, drink (including alcohol), and caffeine on the day of the study.

The study was approved by the Cardiff University School of Psychology ethics committee.

### 2.2 Experimental design and motor task

**Fig. 1.**
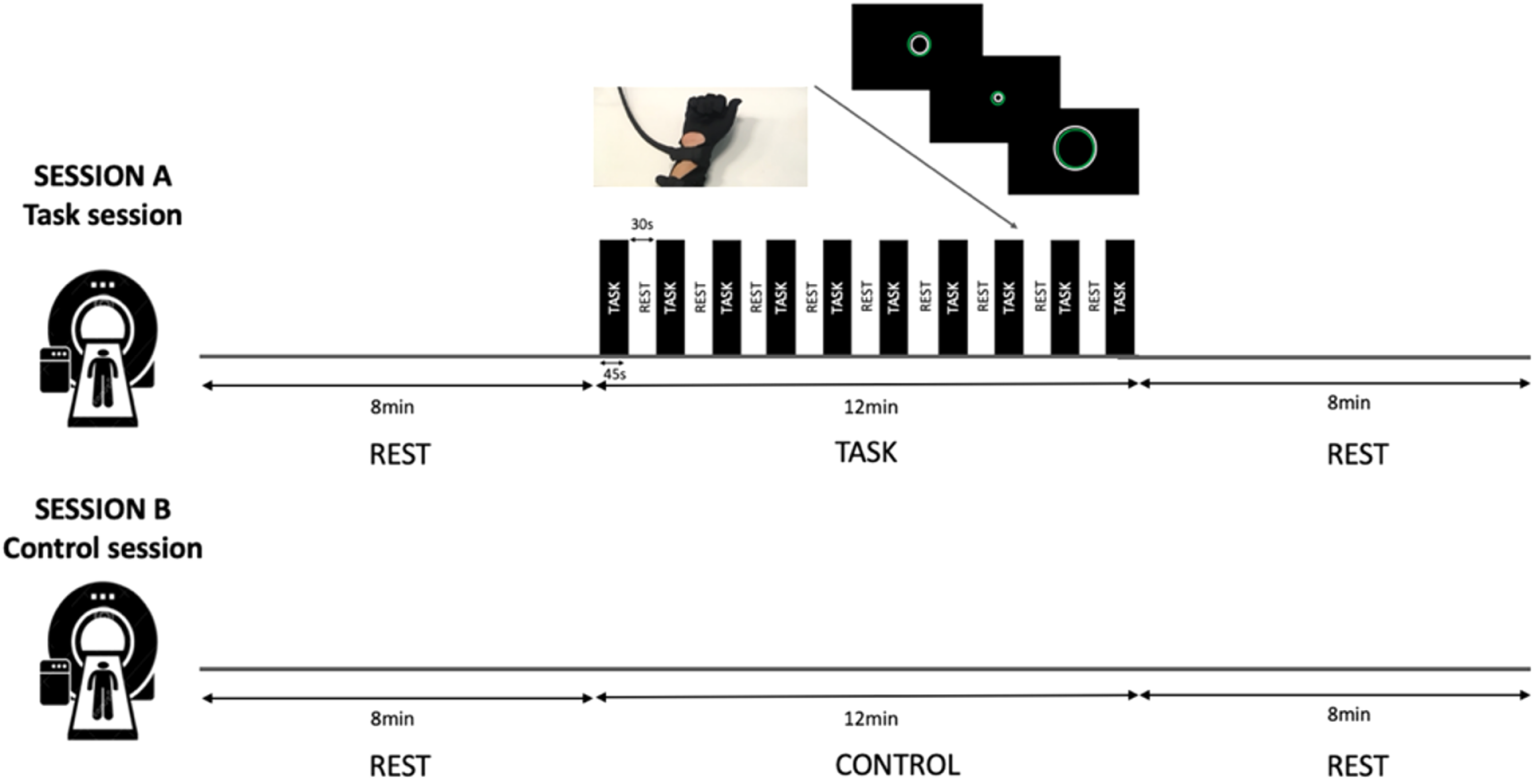
Experimental design. Participants were scanned twice. During the task session (A), participants underwent 8 min resting state, followed by 12 min of motor task, and 8 min of resting state. During the control session (B), participants underwent 8 min resting state, followed by 12 min where they did nothing apart from lying in the scanner and 8 min of resting state again.

In each of the two sessions, participants underwent a structural and a functional MRI scan. During the task session, participants underwent 8-min resting state, followed by 12 min of motor task and 8 min of resting state. During the control session, participants underwent 8 min resting state, followed by 12 min where they did nothing apart from lying in the scanner (effectively a further 12 min of resting state) and 8 min of resting state again (Fig. 1). During the rest scans in the task session and for the entire duration of the control session, a white fixation cross and the word “REST” were presented on a black screen. Ten participants performed the task session first, while the other 10 performed the control session first.

For the present experiment, we used an MRI-compatible right hand data glove (Fifth Dimension Technologies, 5DT Data Glove 16 MRI,https://5dt.com/5dt-data-glove-ultra/) with fibreoptic sensors to measure 14 joint angles of the hand. The data glove was worn by the subjects in the scanner and a set of fibreoptic cables (5 metres long) connected the glove to the control room, where the glove was plugged into the stimulus PC and motor responses were recorded with PsychoPy (Peirce, 2007).

During the task, a white circle presented on a black screen expanded and contracted in a smooth fashion in a fixed sequence. Participants were able to control the size of a green circle, concentric with the white one, by squeezing their hand into a fist. Namely, the green circle changed diameter based on the participant’s flexion and extension of all their fingers. Participants were instructed to continuously match the size of the two circles and they were not told of the existence of a fixed sequence of changes in the diameter of the white circle. The task was split into 10 blocks, each block lasted for 45 s and was followed by a 30 s rest period. During each block, the fixed sequence of size changes was presented three times.

### 2.3 Magnetic resonance imaging

Data were acquired on Siemens Prisma 3T scanner (Siemens Healthineers, Erlangen, Germany), using a 32-channel receive-only head coil. A magnetization prepared rapid acquisition with gradient echo (MPRAGE), T1-weighted structural scan was acquired for registration (1 mm isotropic resolution, 200 slices, TR/TE = 2100/3.24 ms).

All the functional scans were acquired using a pseudo-continuous arterial spin labelling (pCASL) acquisition with pre-saturation and background suppression (Okell et al., 2013) and a dual-excitation (DEXI) readout (Schmithorst et al., 2014) aimed at producing a good ASL signal from the short echo-time data and BOLD contrast from the longer echo-time data. The labelling duration and post-label delay (PLD) were both set to 1.5 s, GRAPPA acceleration (factor of 3) was used with TE1 = 10 ms and TE2 = 30 ms. An effective TR (the total TR including labelling and both readouts) of 4.9 s was used to acquire 16 slices, in-plane resolution 3.4 x 3.4 mm, and slice thickness 7 mm with a 20% slice gap. In total, 175 tag–control pairs resulted in 350 volumes being acquired over the 28-min task, given that the scanner ran continuously through the rest and task periods. A calibration (M0 image) was acquired for ASL quantification with pCASL and background suppression switched off, with TR of 6 s and TE = 10 ms.

During the entire scanning session, physiological monitoring was used to record CO_2_and O_2_end-tidal traces using a nasal cannula connected through a sampling line to a gas analyser system (PowerLab®, ADInstruments, Sydney, Australia).

### 2.4 Data analysis

#### 2.4.1 Behavioural response to the task

To estimate the ability of the subject to accomplish the motor task (continuously match the size of the two circles as closely as possible), a Pearson’s correlation between the time-varying diameter of the white circle (pre-fixed sequence) and the time-varying diameter of the green circle controlled by the participant’s fist (behavioural response) was calculated for each block. To investigate the change in performance during task execution, a repeated measure one-way ANOVA was performed with the correlation index between the circle diameters as the dependent variable and the block number as the independent variable. An increase of correlation over time, from block to block, was taken as a metric of implicit learning of the pre-fixed sequence. All the analyses were conducted with an in-house MATLAB (R2017_b, Mathworks Inc., MA, USA) script.

#### 2.4.2 Task fMRI analysis

The first and second echoes from the pCASL sequence’s data were motion corrected based on six degrees of freedom co-registration method using FSL tool MCFLIRT (Jenkinson et al., 2002) and then brain extracted using BET (Smith, 2002). Spatial smoothing (FWHM = 4.5 mm) of the BOLD data (surround average of TE2) was carried out with SUSAN (Smith & Brady, 1997), with high-pass temporal filter applied with a cut-off time of 90 s. ASL perfusion data (computed employing a surround subtraction of TE1 volumes) were spatially smoothed using a 3D Gaussian kernel (FWHM = 4.5 mm). The FSL tool FLIRT (Jenkinson & Smith, 2001) was used to register BOLD data to individual T1-structural data (12 DOF) and the FSL tool FNIRT (Andersson et al., 2007) was used for non-linear registration of the T1-structural data to MNI standard space (12 DOF). The resulting registration matrix was then used to transform ASL data (first echo) into T1 and MNI spaces using FNIRT (Andersson et al., 2007).

FSL FEAT was used to analyse task fMRI data. BOLD and CBF changes were modelled through a gamma convolution (Woolrich et al., 2004). The BOLD and CBF task responses were modelled for the middle 12-min portion of data: TASK > REST, REST > TASK and linearly decreasing and increasing task activations over time with parametric regressors. MCFLIRT motion correction parameters were added as regressors.

A higher level analysis was performed with FEAT to model the functional responses to the task across all participants using a mixed effects model (FLAME 1). Z statistic images were thresholded at Z > 2.3 with a statistical cluster threshold of p < 0.05.

#### 2.4.3 Changes in resting CBF induced by the motor task

Perfusion maps (pre-processed and transformed to MNI space) were converted into CBF with units of ml/100g/min using the BASIL toolbox (Chappell et al., 2009), assuming a labelling efficiency of 0.85 and T1 of blood = 1.65 s. For each subject, the CBF map of the post-task 8-min rest period was subtracted from the CBF map of the pre-task 8-min rest period. Thus, we obtained maps of CBF change for the task and control scan sessions in standard space. Permutation testing (FSL-RANDOMISE) was used to investigate the differences between the resting CBF change maps between the motor task and control scan sessions at a voxel-wise level. Mean resting end-tidal CO_2_concentration differences between the two resting scans were calculated for each participant and they were included as a covariate in the group level model. Correction for multiple comparisons was performed using threshold-free cluster enhancement (TFCE) (Smith & Nichols, 2009). Differences were considered significant at p < 0.05.

#### 2.4.4 BOLD local connectivity analysis

A regional homogeneity analysis (ReHo) (Zang et al., 2004) was used to investigate the effect of the task on reorganization of local connectivity (often assumed to be related to local activity) in areas showing a significant increase in CBF during the resting period after the task.

Pre-processed BOLD time series were band-pass filtered (0.01-0.1 Hz) using FSL and then transformed to individual T1-structural data and to MNI standard space using FNIRT (Andersson et al., 2007) using the resulting registration matrix from the above pre-processing. Motion correction parameters (based on six degrees of freedom co-registration method) and physiological parameters (end-tidal CO_2_) were regressed from the image timeseries data using SPM12 (www.fil.ion.ucl.ac.uk/spm).

ReHo was calculated on the post-processed BOLD MRI images using MATLAB (Mathworks Inc.) toolbox DPABI V5.3 (Yan et al., 2016), in subject space via Kendalls coefficient of concordance (KCC) as a local coherence metric of BOLD signal, with 27 neighbouring voxels, for each resting period (pre-task, post-task, pre-control, post-control) of the experiment. ReHo maps were then spatially z-scored to perform the analysis. Differences between post and pre (ΔReHo) value were computed for each session (task and control). A paired one-tailed*t*-test was employed to assess whether ΔReHo metrics increased after motor task execution compared with the control session, in areas demonstrating a significant increase in CBF during the resting period after the task. Differences were considered significant at p < 0.05.

#### 2.4.5 BOLD functional connectivity analysis

A functional connectivity (FC) analysis was performed using the processed BOLD fMRI images to investigate whether sustained CBF changes in the period after completion of the task are also associated with changes in FC. Areas showing a significant increase in CBF during the resting period after the task were used as a seed region to investigate changes in FC between that seed region and 116 regions of interest (ROIs) (covering the entire brain) pre-defined in MNI space by the Automatic Anatomical Labelling Atlas (AAL) (Tzourio-Mazoyer et al., 2002). For each subject, the mean (BOLD) timeseries across voxels was extracted for the seed region and each of the 116 ROIs using FSL. Analysis was performed in the standard MNI space. In order to assess the strength of FC, correlation coefficients were calculated, for each of the four resting periods (pre/post task, pre/post control), by correlating the mean time course of the seed region with the mean time course of each ROI.

A Fisher-z transformation was employed to transform the sampling distribution of the correlation coefficient to make it normally distributed. A repeated measure two-way ANOVA was performed on z-values to investigate the differences in FC pre/post the task compared with the control session. Differences were considered significant if p < 0.05. The analyses were performed on Rstudio (http://www.rstudio.com/).

## 3 Results

### 3.1 Motor task

The ANOVA demonstrated a main effect of block (time) (F_(9,171)_= 10.89, p < 0.001). As shown inFigure 2, there was an increase in temporal correlation of the diameter of the two circles from block to block and so an improvement of performance with time. Most of the improvement happened in the first three blocks after which performance remained largely stable.

**Fig. 2.**
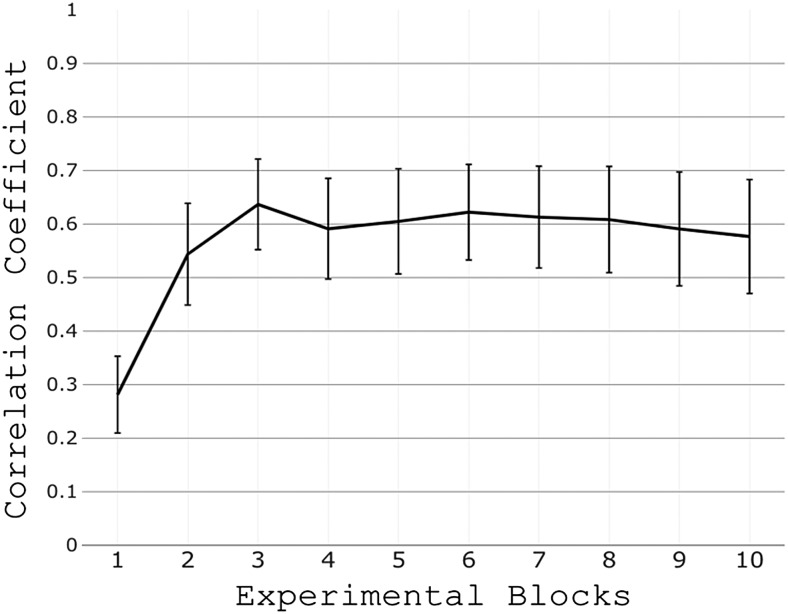
Behavioural results. Increasing temporal correlation between the time-dependant diameters of the two circles throughout task performance (mean ± SEM across participants). This result suggests an improvement in performance especially over the first three blocks.

### 3.2. Task-related fMRI responses

**Fig. 3.**
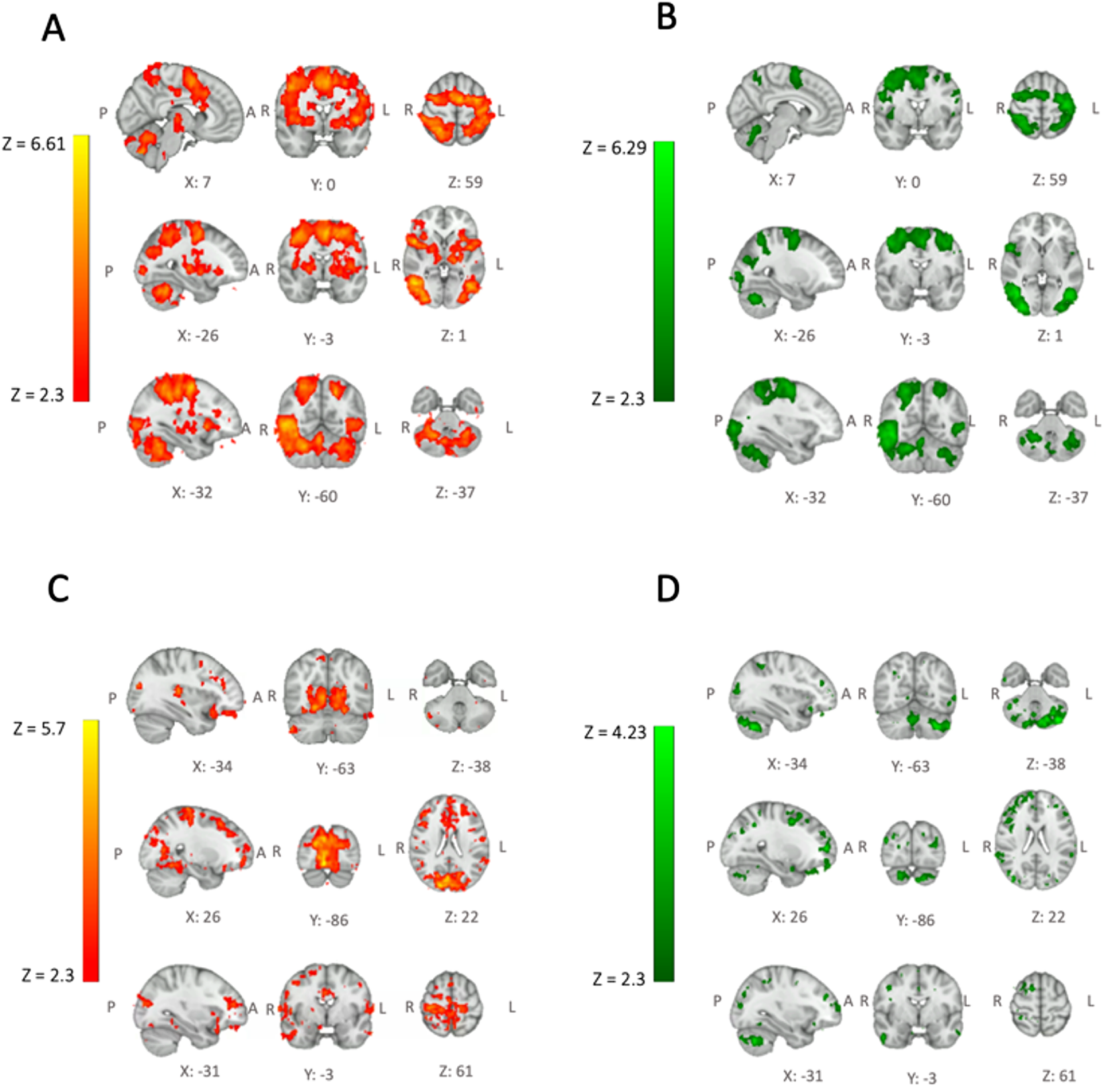
Top Row: Task execution responses. BOLD (A) and CBF (B) mean responses to task execution reported as z stats. Main areas of BOLD and CBF responses to the task were found bilaterally in post-central gyri, inferior occipital cortex, superior parietal lobules, cerebellum (lobules V–VI), and left pre-central gyrus. BOLD activation was also found bilaterally in putamen and in left thalamus. Bottom Row: Reductions of task responses. Areas of linearly decreasing BOLD (C) and CBF (D) task response across blocks reported as z statistics. Z statistic images were thresholded at Z > 2.3 with a two-sided statistical cluster threshold of p < 0.05.

Main areas of positive BOLD and CBF responses during the task (task > rest) were found bilaterally in post-central gyri, inferior occipital cortex, superior parietal lobules, cerebellum (lobules V–VI), and left pre-central gyrus. Significant positive BOLD responses were also found bilaterally in putamen and in left thalamus (Fig. 3A and B). Areas of negative BOLD and CBF responses (rest > task) were found bilaterally in the cuneal/cingulate cortex and in the frontal medial cortex (indicating the default mode network activation during the rest periods between the task blocks).

Regions of significant linear reduction from block to block of task-induced BOLD response were observed mainly bilaterally in pre-central gyri. Regions of significant linear reduction of task-induced CBF response were observed mainly bilaterally in cerebellum (Fig. 3CandD). Regions of significant linear increase of task-induced BOLD response were observed bilaterally in the orbital cortex, left pre-cuneal cortex, and medial cingulate gyrus. No such increases were observed for the task-induced CBF response.

### 3.3 Sustained increase in CBF after task execution

**Fig. 4.**
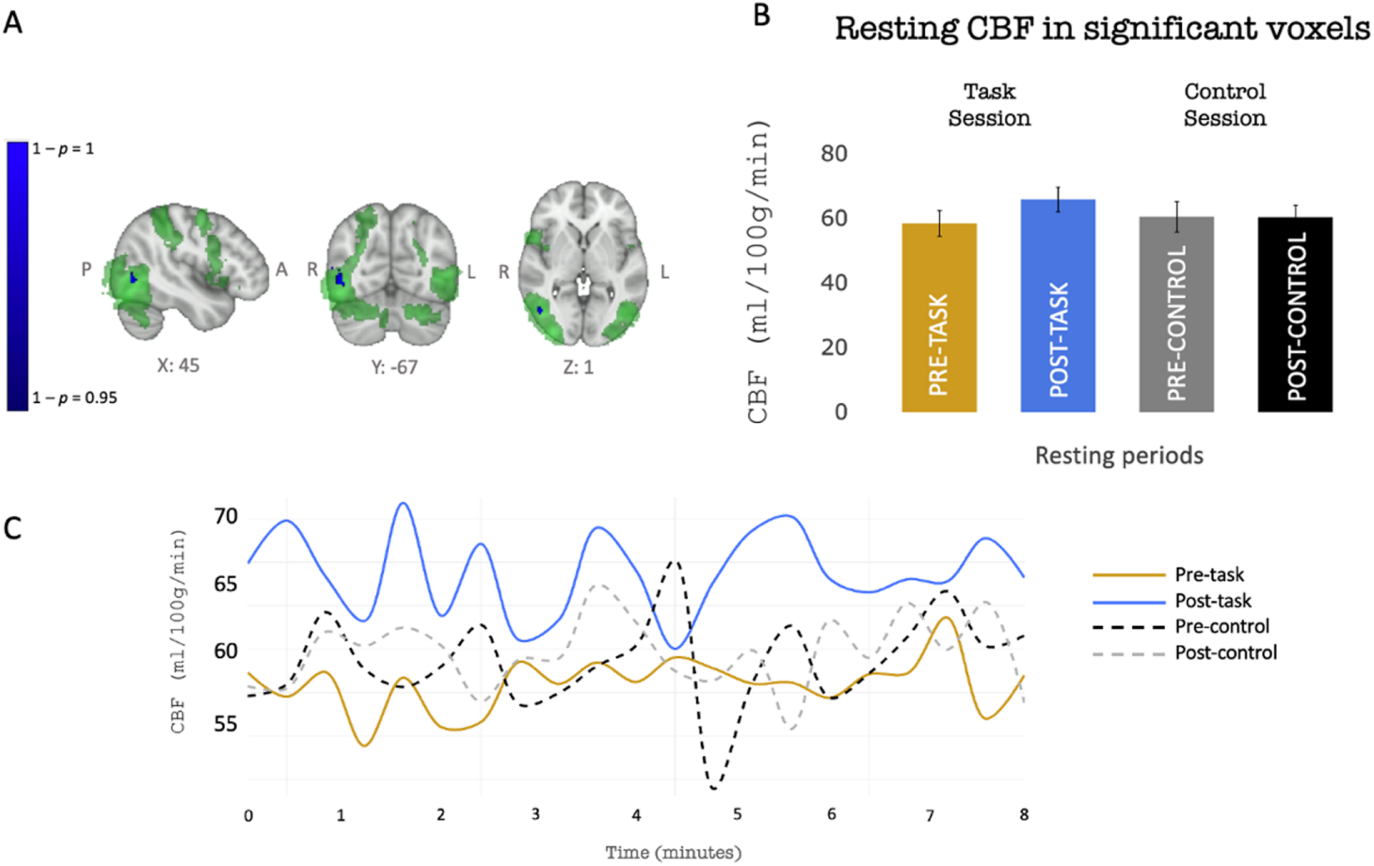
Top Row: Sustained CBF increase following the completion of the task (group level). (A) Blue: Area (MT/V5) showing increase in resting CBF (blue) after task execution (reported as p value). This area constituted our region of interest (ROI) for BOLD functional connectivity analysis. Green indicates mean BOLD-related task response across the 10 experimental blocks reported as z stats. Z statistic image was thresholded at Z > 2.3 with a two-sided statistical cluster threshold of p < 0.05**.**(B) Group mean ± SEM resting CBF during the different resting periods (pre-/post- task/control) in the region of increased CBF indicated in (A). Bottom Row: Group mean CBF during the different rest periods in area (MT/V5). (C) For each resting-state period (pre/post task, pre/post control), CBF (y axis) in significant area (MT/V5 as shown in A) was plotted over the duration of the rest acquisition (x axis). Blue line represents CBF after the task showing a higher CBF compared with the other rest periods for the entire duration of the measurement (8 min). This suggests that the increased CBF response is sustained for at least 8 min after the end of the task.

Voxel-wise analysis showed a localized increase in perfusion in the rest period after task compared with before the task only in the session in which the motor task was performed and not in the control session. Significantly increased perfusion after the task was found in right extra-striate visual area (MT/V5), this area was also implicated in task performance (Fig. 4A). A plot of CBF over time in each of the 8-min rest periods (pre-task, post-task, pre-control, post-control) suggests that the local increase in CBF was sustained at least for the entire duration of the rest acquisition (8 min after the end of the task) (Fig. 4C). We did not observe a decrease of CBF after the completion of the task in any regions of the brain.

### 3.4 Local functional connectivity

**Fig. 5.**
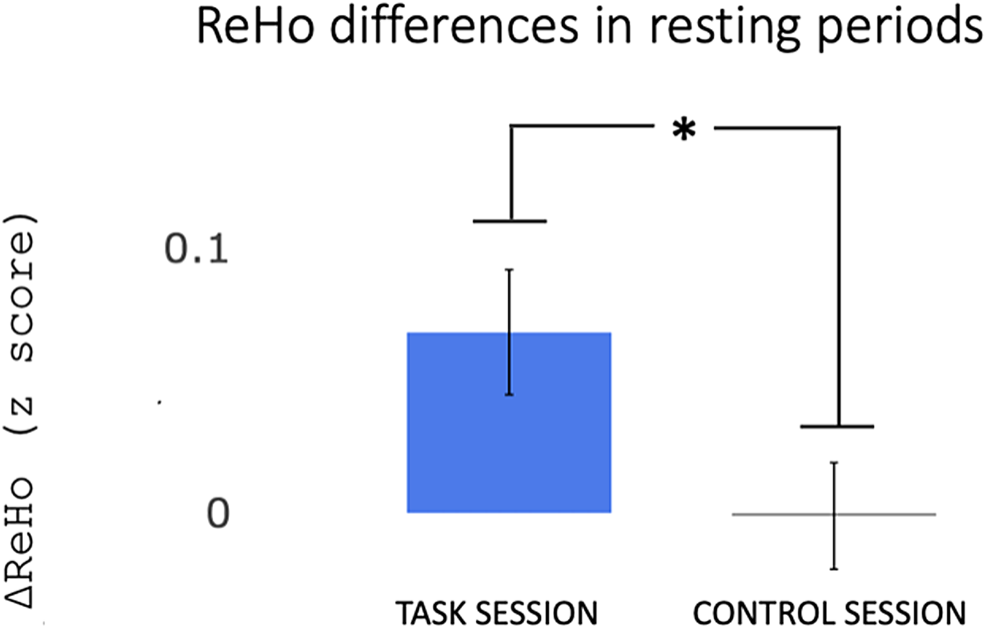
Differences in ReHo metrics at each experimental session. ReHo metrics were calculated for each resting period (pre-task, post-task, pre-control, post-control) in the MT/V5 area, identified as exhibiting raised CBF, and converted to z-scores. Differences in each session are plotted (blue: ReHo differences between post-task and pre-task session; grey: ReHo differences between post-control and pre-control session). The values are plotted as mean and SEM. An increase of local connectivity (ReHo) in the resting period after the execution of the task (blue) was observed. The increase was not observed in the control session (grey). Statistical significance was assessed using a one-tailed*t*-test, denoted by an asterisk (*) to indicate a significance level of p < 0.05.

ReHo values were calculated for each resting period (pre-task, post-task, pre-control, post-control) in the area of MT/V5 identified as exhibiting a sustained increase in CBF (area shown inFig. 4A), and the differences between post and pre (ΔReHo) for each session (task and control) were computed. A*t*-test showed higher ΔReHo in the task session compared with the control session (t_(19)_= 1.81, p = 0.04), indicating that ReHo increased only after performing the motor task in MT/V5 (Fig. 5).

### 3.5 Long-range functional connectivity

Correlation coefficients (as an index of functional connectivity) between the seed area shown inFigure 4A(MT/V5 - area of elevated CBF) and each ROI (116 areas defined on the AAL) were calculated for each subject for each of the four 8-min rest periods on the BOLD maps. The correlation coefficients are reported inTable S1(seeSupplementary Material) as mean ± SEM across subjects for each time point.

The ANOVA showed no main effects of time (before or after task) or session (motor task vs control) and no interaction effect (time x session) in any of the ROIs indicating that seed-ROI BOLD-FC did not change in the second rest period compared with the first, and it did not change with session (motor task vs control). The matrices ofFigure S1(seeSupplementary Material) show the correlation index (FC) and their significance (p value) for each seed-ROI correlation index (Z score transformed) at each time point.

## 4 Discussion

We have demonstrated a localized CBF increase in MT/V5 in the 8 min after the completion of a 12-min motor learning task accompanied by an increased synchronization of local BOLD signal. We also demonstrated decreasing BOLD and CBF responses during task execution, accompanied by an improvement in performance, suggesting that our task was well designed to probe effects of motor learning and thus potential neuroplasticity.

### 4.1 A data glove as a new tool to study neuroplasticity

To the best of our knowledge, we have demonstrated for the first time the use of a data glove to probe neuroplasticity in healthy volunteers. Previous studies have employed a data glove in the context of neurorehabilitation, brain recovery, and assessment of functions in clinical populations (Bonzano et al., 2015;Limanowski et al., 2017;Ranganathan, 2017;Richards et al., 2015), whereas we asked participants to perform a sequence learning task. Neuroimaging studies have largely employed variants of the serial reaction time task (SRTT) (Nissen & Bullemer, 1987) to study neural correlates and adaptations of sequential motor learning (Hardwick et al., 2013). The brain areas commonly showing activity in such tasks include the striatum, cerebellum, and pre-frontal areas (Robertson, 2007), while performance improvements have been associated with activation in pre-motor cortical regions, caudate, and associative cerebellar regions (Hikosaka et al., 2002). Our results are consistent with the above studies.

We also observed a decrease of activation between blocks during task execution which is consistent with previous studies reporting that motor training is associated with reduced excitability of the motor cortex (Pascual et al., 1995;Shadmehr & Holcomb, 1997). Decreasing activity revealed during fMRI studies may reflect changes due to increased efficiency, reduced errors, and consolidation of learning (Hardwick et al., 2013). We found a decrease in both BOLD and CBF modulations, indicating a change in the recruitment of neural resources (Reber, 2013).

### 4.2 Baseline perfusion increases following 12 min of motor task

The localized increase in CBF sustained after the motor task was specific to the task as it was not observed following a rest period of equivalent duration. It is not, therefore, explicable by the simple passage of time. To the best of our knowledge, the present study is the first to investigate changes in resting CBF in the minutes after the execution of a motor task compared with a period of rest before task execution. Previous studies have used BOLD fMRI to assess functional changes following task execution and they demonstrated that experience-dependent sensorimotor plasticity can induce changes in BOLD RS signal fluctuations (Amad et al., 2017;Ge et al., 2015;Taubert et al., 2011;H. Zhang et al., 2014). Such studies have shown that resting-state BOLD fMRI offers a valuable index of the functional changes that support sensorimotor adaptive processes (Guerra-Carrillo et al., 2014). In particular, motor training studies show that the changes in BOLD RSN are not confined to canonical regions supporting motor functions (Guerra-Carrillo et al., 2014). Therefore, motor learning can induce changes in FC, and this is suggestive of a functional reorganization of the brain networks supporting the offline consolidation of new motor skills (Sami et al., 2014). Furthermore, changes in spontaneous brain fluctuations have been associated with the performance improvement (Vahdat et al., 2011). We observed changes in perfusion in the right extra-striate visual area (MT/V5). Previous studies have shown that MT/V5 is activated during visual motion processing (Brandt et al., 2000;Peuskens et al., 2001) and it correlates with the ability of the subject to pay attention to the visual motion and complete the task (Büchel et al., 1998;Friston & Büchel, 2000), therefore, it is involved in visual processing task (Dupont et al., 1994;Olson et al., 2004). The attentional component of MT/V5 is also supported by the extensive connectivity with lower attentional areas. MT/V5 is anatomically connected with subcortical regions such as claustrum, putamen, and thalamic nuclei (Maunsell & Van Essen, 1983), which are regions that are widely involved in motor learning (Doyon et al., 2003;Lehéricy et al., 2005). Furthermore, previous studies that used BOLD signal to investigate RS changes after tasks/training report modifications in areas involved in the process of motor learning (Albert, Robertson, Miall, et al., 2009;Amad et al., 2017;H. Zhang et al., 2014).

We suggest that the observed changes in perfusion, sustained after the completion of the task, mark the underlying reorganization that supports motor skill acquisition. However, we cannot identify from the present experiment the underlying causes of the CBF increase, the principal possibilities being: (i) sustained increase in neuronal activity with concomitant increase in metabolic demand; (ii) preparatory adaptation of microvascular function without a concomitant increase in metabolic demand, and (iii) the time needed for a return to CBF baseline levels.

Considering the first possibility and given the link between CBF and glucose utilization/oxygen consumption (Raichle et al., 2001;Vaishnavi et al., 2010), the increase in perfusion may reflect a higher metabolic demand in that area associated with activation of specific neuronal networks and release of messengers responsible for neurovascular coupling (Lecrux et al., 2019;Mathias et al., 2018). Although the identification of the precise neural correlates of haemodynamic responses is complex, it has been observed that changes in haemodynamic responses correlate with changes in gamma band (Harris et al., 2014;Niessing et al., 2005;Shmuel & Leopold, 2008), which are a signature of engaged neuronal networks (Jia & Kohn, 2011). Overall, we can speculate that sustained CBF responses in specific areas could be attributed to a greater engagement of the underlying networks.

Considering the second possibility, cerebrovascular plasticity is observed at many hierarchic levels and the timescale of these different processes spans a wide range. Changes in CBF in response to increases in neural activity occur with a time constant on the order of seconds, whereas changes in cerebrovascular architecture can be due to sensory deprivation, aerobic exercise, or hypoxia, and they can occur with time constants of several weeks (Bogorad et al., 2019). It is not known, but it remains possible, that the local microvascular network begins its functional and/or structural adaptation to the longer term need to increase CBF within minutes of the completion of a learning task. It has been reported that physical exercise leads to global and regional CBF changes in both healthy subjects and people with vascular disease (Bliss et al., 2021;Stillman et al., 2020). The neurobiological link between exercise and plasticity is the brain-derived neurotrophic factor (BDNF). Physical exercise increases the expression of BDNF which is involved in neuronal plasticity, neurogenesis, synaptogenesis, hippocampal, and cognitive function (Bliss et al., 2021;Stillman et al., 2020). A similar biological mechanism may be at work during motor task training.

Given the temporal proximity of the observed changes in CBF to the cessation of the task, it may be more likely that CBF increase after the task is caused by changes in microvascular function or changes in neuronal activity, rather than microvascular remodelling.

Considering the third possibility, it is known that the oxygen-glucose index (OGI) at rest is approximately 6.0 (Dalsgaard, 2006) and it falls rapidly during task activation; indicating a higher consumption of glucose upon stimulus activation. This implies that an increased proportion of ATP production is provided by aerobic glycolysis (Vaishnavi et al., 2010). The recovery to baseline OGI is slow after the task ends (Dienel & Cruz, 2016), and during this time, CBF may remain elevated to supply the energy needed to the tissue. Additionally, a PET study (Vafee et al., 2012) confirms previous evidence that activity-related increases of blood flow are rarely accompanied by proportional increases of oxygen consumption (Fox et al., 1988;Fujita et al., 1999;Gjedde et al., 2002), demonstrating that prolonged brain activation leads to glycolytic energy supply while the task is still ongoing, and OGI increases again after 20 min of task execution.

Although their measurements differ from ours, as they were taken while subjects were still performing the task, we may still consider the hypothesis that our temporal window was not long enough to detect a return to baseline OGI values. Therefore, the observed elevation in resting CBF after the completion of the task may be a proxy of ongoing aerobic glycolytic processes (Shannon et al., 2016). A better investigation of the flow-metabolism coupling after task execution is, therefore, needed.

Our three explanations for the observed sustained CBF are not mutually exclusive, as sustained neural activity needs to be supported by a switchover of metabolic support, which, in the long term, may lead to vascular re-modelling. Further longitudinal studies are needed to test the concomitance of these different events.

ASL signal encodes the time course of a single physiological parameter (CBF) and it can detect changes in a lower frequency range (Borogovac et al., 2010) compared with BOLD signal which is dominated by noise at low frequency. Furthermore, CBF measurements are highly reproducible across time and scanners (Jann et al., 2015). We can conclude that CBF is an important imaging marker for characterizing resting brain function and for understanding better the neurovascular activity underlying resting states.

### 4.3 Local effect of motor task intervention

The sustained CBF in MT/V5 was also accompanied by an increase of ReHo in that region, indicating that the execution of the 12-min motor task leads to increased regional synchronization of neural activity as measured by spontaneous low-frequency BOLD signals. Given the high test–retest reliability of ReHo (Zuo et al., 2013;Zuo & Xing, 2014) and its ability to detect regional changes (Zang et al., 2004), it has been proposed that ReHo could be a complementary powerful method to investigate regional plasticity (Zheng et al., 2015), and our results support this idea. Furthermore, our results are in line with a previous study reporting ReHo variations associated with individual improvements in cognitive performance during longitudinal training (Zheng et al., 2015).

The observed increase in CBF accompanied by enhanced ReHo is an argument in favour of ReHo as an fMRI metabolic proxy. Studies investigating the coupling between ReHo and brain metabolic rates report a positive association between metabolic measures and ReHo (Bernier et al., 2017;S. Deng et al., 2022). It is known that high-energy utilization relies in part on higher perfusion, as reported by PET studies where a robust association between brain metabolic rate of glucose and CBF is shown (Aanerud et al., 2012;Huisman et al., 2012). Hence, enhanced CBF may contribute to a higher level of brain metabolic rate of glucose, as well as increase in ReHo. This hypothesis is further supported by previous studies reporting a positive correlation between ReHo and CBF (Li et al., 2012;Zou et al., 2009), indicating that CBF might, to some extent, contribute to the explanation of ReHo (Bernier et al., 2017).

Together the previous studies converge on the idea that the BOLD signal at rest is believed to reflect synaptic activity (Ekstrom, 2010) and this activity results in changes in cerebral metabolic rate (Shannon et al., 2016). The observed concurrent increase in CBF alongside ReHo changes is more likely attributable to changes in microvascular function or neural activity, rather than microvascular remodelling.

### 4.4 The sustained CBF response is not a proxy of BOLD functional connectivity changes

The hypothesis for the present study, that motor learning causes CBF changes after task completion, was born from previously published observations of changes in BOLD FC sustained after the completion of a motor task. Previous studies have described the resting state after a task as a “task-driven” functional network state (Wang et al., 2012) associated with learning and cognition (Albert, Robertson, Miall, et al., 2009;Grigg & Grady, 2010;Hasson et al., 2009;Lewis et al., 2009;Vincent, 2009;Waites et al., 2005). The results have been interpreted as subjects covertly rehearsing the task they have just completed, given that they were interested in improving their performance. The process of rehearsing engages the task-dependent brain network dynamics just evoked and promotes learning and adaptation during this rest period. Such recruitment of task-related brain dynamics during a post-task resting state might facilitate the development of task-related expertise (Muraskin et al., 2016;H. Zhang et al., 2014). Resting-state fMRI is then considered an effective measure of plasticity where the activity patterns can reflect the history of repeated activation between and within regions (Amad et al., 2017), and previous studies reported strengthening of connections assessed with BOLD after motor tasks of various difficulties (Lewis et al., 2009;Tung et al., 2013), observed also after a few minutes following the training (Albert, Robertson, Mehta, et al., 2009;Tambini et al., 2010;Waites et al., 2005).

To test whether changing BOLD FC is linked to the observed alteration in CBF, we examined the BOLD FC between the region of increased CBF in right extra-striate visual area (MT/V5) and the rest of the brain on a region-wise basis. However, we observed no such changes in FC (Fig. S1). This observation suggests that CBF mapping provides different information concerning changes in neural network activity compared with BOLD FC analysis. This is not surprising given that we expect CBF over 8 min to mark tonic changes in neuronal activity, whereas BOLD FC is sensitive to fluctuations over a shorter timescale. The simultaneous acquisition of both BOLD FC and CBF ASL data may provide complementary information about brain functional network reorganization and potentially related underlying neurovascular changes. We note that the manner in which BOLD fMRI data were collected in the present study was not optimized for ReHo or FC analysis. Specifically, the TR was long and the voxel size was large, both constraints imposed by the need to use ASL to address our primary hypothesis regarding CBF changes. Despite the long TR, we were able to investigate the typical frequency range of BOLD fluctuations used in ReHo and FC correlation analysis (0.01–0.1 Hz), with the maximum frequency evaluated below the Nyquist frequency of recordings. As a result of the limited number of samples, we anticipate that the longer TR reduces the confidence in our estimates, but provided an unbiased estimation compared with what we would have been obtained with a shorter TR and the same explored signal frequencies.

However, it is important to highlight that the reduced spatial resolution is indeed a limitation of our study. This limitation could introduce bias in the estimation of ReHo and FC, both influenced by partial volume effects and the inherently coarser estimation of temporal signal correlations across space.

Moreover, the lack of significant findings in relation to FC between MT/V5 and the rest of the brain does not exclude the possibility that FC changes occur between other brain regions.

## 5 Conclusion

We have demonstrated a localized change in resting CBF and BOLD ReHo in right extra-striate visual area (MT/V5) during a period of rest after the completion of a motor learning task. This may be suggestive of ongoing mechanisms related to neuronal network plasticity either from sustained alterations of neuronal activity or from modification of the functional status of the local microvasculature. Further studies are needed to isolate the neurovascular mechanisms underlying reorganization after the competition of the motor task.

## Data and Code Availability

The data supporting the conclusions of this study are available athttps://doi.org/10.17035/cardiff.26739589. Access to the dataset can be obtained by contacting the corresponding author, or by following the repository guidelines.

## Author Contributions

E.P., R.G.W., and V.T. designed research; E.P. performed research; M.G. assisted in research; E.P. and D.D.C. analysed data; E.P., D.D.C, A.M.C., and R.G.W. wrote the paper.

## Funding

This work was supported by Wellcome Trust Ph.D. studentship [203965/Z/16/Z] and partially supported by the UK Engineering and Physical Sciences Research Council (EP/S025901/1).

This research was funded, in part, by the Wellcome Trust Strategic Award [104943/Z/14/Z] and Sir Henry Dale Fellowship [220575/Z/20/Z].

This work was partially conducted under the framework of the Departments of Excellence 2023–2027 initiative of the Italian Ministry of Education, Universities and Research for the Department of Neurosciences, Imaging and Clinical Sciences (DNISC) of the University of Chieti-Pescara, Italy.

Funded, in part, by the European Union—NextGenerationEU under the National Recovery and Resilience Plan (NRRP), Mission 4 Component 2—M4C2, Investment 1.5—Call for tender No. 3277 of 30.12.2021 Italian Ministry of Universities Award Number: ECS00000041, Project Title: “Innovation, digitalisation and sustainability for the diffused economy in Central Italy,” Concession Degree No. 1057 of 23.06.2022 adopted by the Italian Ministry of Universities, CUP: D73C22000840006.

Funded, in part, by the European Union—NextGenerationEU—Italian Ministry of University and Research (MUR), Research National Program (PNR) and Projects of National Relevance (PRIN), Project Code: 2022BERM2F, Project Title: “Mapping Mitochondrial Function and Oxygen Metabolism in the Human Brain with Magnetic Resonance Imaging.” Funding call No. 104 of 02.02.2022, Concession decree No. 1065 of 18.07.2023 adopted by MUR, ERC Panel LS7 “Prevention, Diagnosis and Treatment of Human Diseases”. CUP: D53D23013410001.

Funded, in part, by the European Union—NextGenerationEU—Italian Ministry of University and Research (MUR), National Plan for Recovery and Resilience (PNRR) and Projects of National Relevance (PRIN), Project Code: P20225AEEE, Project Title: “Hybrid PET-MRI to simultaneously probe brain metabolism and cerebrovascular function in neurodegenerative diseases.” Funding call No. 1409 of 14.09.2022, Concession decree No. 1369 of 01.09.2023 adopted by MUR, ERC Panel LS7 “Prevention, Diagnosis and Treatment of Human Diseases”. CUP: D53D23021480001.

Funded, in part, by the European Union—NextGenerationEU—Italian Ministry of University and Research (MUR), National Plan for Recovery and Resilience (PNRR) and Projects of National Relevance (PRIN), Project Code: P2022ESHT4, Project Title: “Advancing MRI biomarkers of brain tissue microstructure and energetics in Multiple Sclerosis.” Funding call No. 1409 of 14.09.2022, Concession decree No. 1367 of 01.09.2023 adopted by MUR, ERC Panel LS5 “Neuroscience and Disorders of the Nervous System”. CUP: D53D23019210001

For the purpose of open access, the author has applied a CC BY public copyright license to any Author Accepted Manuscript version arising from this submission.

## Declaration of Competing Interest

Authors declare that the research was conducted in the absence of any commercial or financial relationships that could be construed as a potential conflict of interest.

## Supplementary Materials

Supplementary material for this article is available with the online version here:https://doi.org/10.1162/imag_a_00282.

## Supplementary Material

Supplementary Material
