# Supplementary material for: Evidence for a sustained cerebrovascular response following motor practice
Source: Imaging Neurosci (Camb). 2024 Aug 29;2:imag-2-00282. doi: 10.1162/imag_a_00282 (PMC12290683; doi:10.1162/imag_a_00282)
Supplement: Supplementary Material [file imag_a_00282-supp.pdf]

Supplementary Material

METHODS

Sustained CBF and performances. To investigate the relationship between changes in CBF after task execution and the slope of behavioural performance improvement, we conducted a Pearson correlation analysis using Rstudio (http://www.rstudio.com/). Correlation were considered significant at $p < 0.05$.

RESULTS

Sustained CBF and performances. We did not observe a significant correlation between changes in CBF after task execution and the slope of behavioural performance improvement ($R=0.07; p=0.77$).

Functional Connectivity.

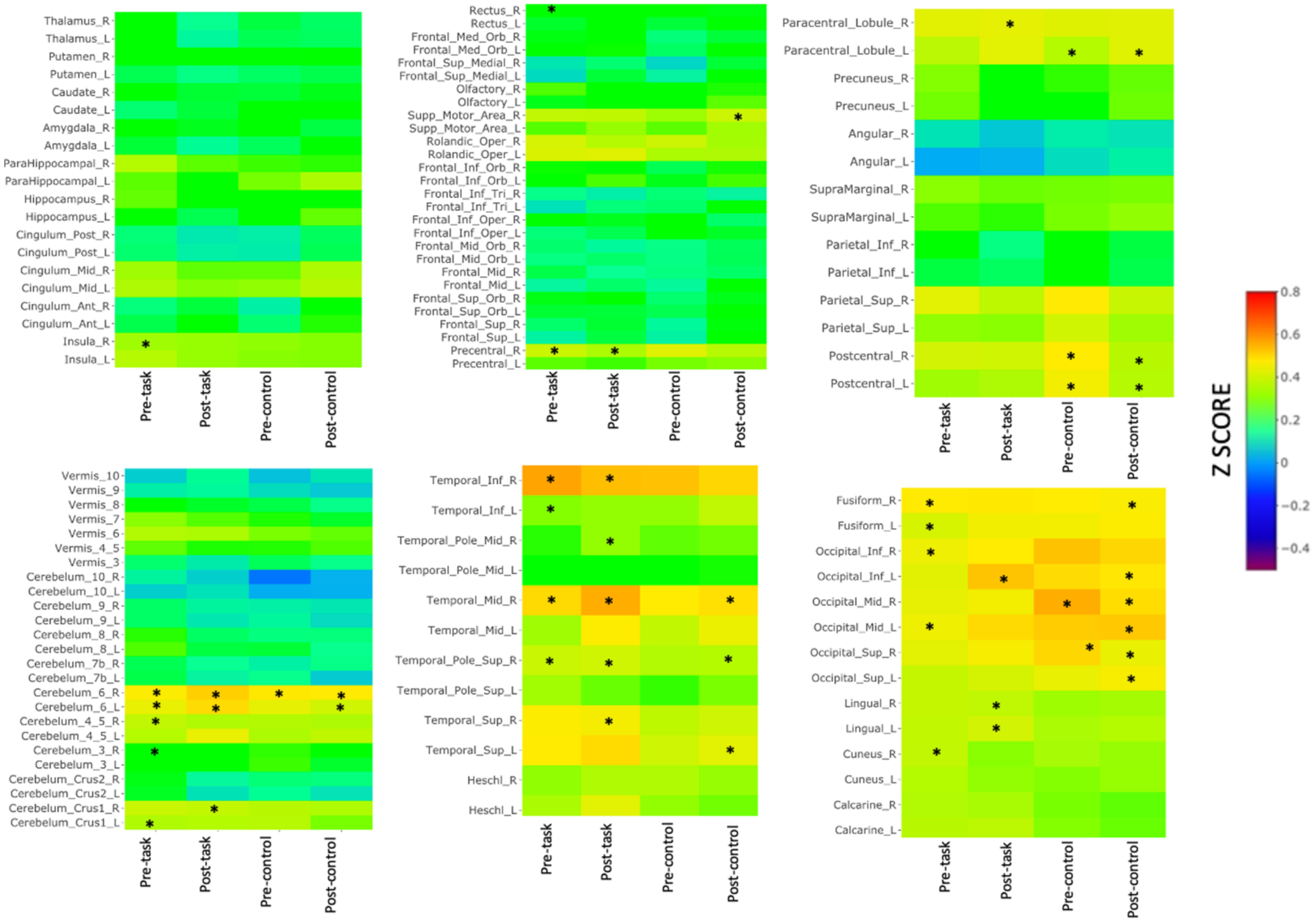

Fig. S1. ***Seed-ROI functional connectivity matrix***. *The matrix shows the correlation indices (FC - Z score transformed) and their significance (p value) for each seed-ROI pair at each time point. * indicates significant correlations at p < 0.05.*

Table S1. ***Seed-ROIs correlation coefficients.*** *Correlation coefficients reported for each ROI at each resting period (before/after task) and in each session (motor task/control) as Mean±SEM.*

Seed-ROI correlation coefficients (mean ± sem)

	Pre-Task	Post-Task	Pre-Control	Post-Control
**Precentral_L**	0.31±0.25	0.29±0.3	0.32±0.29	0.34±0.29
**Precentral_R**	0.41±0.27	0.38±0.31	0.43±0.26	0.39±0.26
**Frontal_Sup_L**	0.12±0.23	0.21±0.31	0.12±0.27	0.27±0.27
**Frontal_Sup_R**	0.18±0.24	0.21±0.27	0.13±0.28	0.23±0.28
**Frontal_Sup_Orb_L**	0.21±0.25	0.21±0.24	0.2±0.32	0.26±0.32
**Frontal_Sup_Orb_R**	0.23±0.22	0.23±0.3	0.17±0.31	0.22±0.31
**Frontal_Mid_L**	0.13±0.26	0.18±0.3	0.13±0.29	0.26±0.29
**Frontal_Mid_R**	0.2±0.25	0.14±0.32	0.16±0.31	0.18±0.31
**Frontal_Mid_Orb_L**	0.16±0.23	0.18±0.27	0.15±0.29	0.19±0.29
**Frontal_Mid_Orb_R**	0.17±0.22	0.13±0.31	0.15±0.28	0.19±0.28
**Frontal_Inf_Oper_L**	0.17±0.2	0.2±0.3	0.25±0.29	0.22±0.29
**Frontal_Inf_Oper_R**	0.24±0.28	0.22±0.36	0.27±0.3	0.18±0.3
**Frontal_Inf_Tri_L**	0.1±0.22	0.16±0.31	0.18±0.27	0.23±0.27
**Frontal_Inf_Tri_R**	0.15±0.25	0.12±0.37	0.17±0.33	0.12±0.33
**Frontal_Inf_Orb_L**	0.24±0.21	0.3±0.27	0.22±0.25	0.29±0.25
**Frontal_Inf_Orb_R**	0.28±0.22	0.25±0.34	0.19±0.28	0.28±0.28
**Rolandic_Oper_L**	0.43±0.23	0.43±0.24	0.38±0.27	0.38±0.27
**Rolandic_Oper_R**	0.42±0.27	0.39±0.3	0.41±0.27	0.37±0.27
**Supp_Motor_Area_L**	0.3±0.25	0.36±0.27	0.31±0.23	0.37±0.23
**Supp_Motor_Area_R**	0.4±0.24	0.4±0.25	0.36±0.22	0.41±0.22
**Olfactory_L**	0.22±0.22	0.25±0.25	0.25±0.25	0.31±0.25
**Olfactory_R**	0.3±0.24	0.26±0.26	0.26±0.3	0.28±0.3
**Frontal_Sup_Medial_L**	0.09±0.25	0.21±0.33	0.12±0.29	0.27±0.29
**Frontal_Sup_Medial_R**	0.1±0.26	0.17±0.33	0.08±0.29	0.21±0.29
**Frontal_Med_Orb_L**	0.24±0.26	0.28±0.27	0.18±0.31	0.26±0.31
**Frontal_Med_Orb_R**	0.23±0.27	0.25±0.25	0.16±0.3	0.21±0.3

**Rectus_L**	0.22±0.25	0.23±0.24	0.21±0.32	0.23±0.32
**Rectus_R**	0.23±0.27	0.24±0.22	0.25±0.31	0.23±0.31
**Insula_L**	0.39±0.23	0.35±0.21	0.34±0.27	0.34±0.27
**Insula_R**	0.36±0.29	0.35±0.23	0.35±0.24	0.34±0.24
**Cingulum_Ant_L**	0.2±0.28	0.25±0.26	0.17±0.28	0.28±0.28
**Cingulum_Ant_R**	0.16±0.28	0.21±0.26	0.12±0.26	0.23±0.26
**Cingulum_Mid_L**	0.38±0.29	0.34±0.23	0.35±0.24	0.39±0.24
**Cingulum_Mid_R**	0.36±0.29	0.31±0.25	0.31±0.24	0.38±0.24
**Cingulum_Post_L**	0.17±0.28	0.12±0.33	0.11±0.31	0.19±0.31
**Cingulum_Post_R**	0.17±0.27	0.11±0.32	0.12±0.3	0.19±0.3
**Hippocampus_L**	0.27±0.19	0.2±0.26	0.25±0.26	0.31±0.26
**Hippocampus_R**	0.31±0.22	0.25±0.25	0.24±0.24	0.25±0.24
**ParaHippocampal_L**	0.31±0.19	0.27±0.28	0.32±0.24	0.38±0.24
**ParaHippocampal_R**	0.38±0.19	0.31±0.25	0.29±0.29	0.28±0.29
**Amygdala_L**	0.21±0.18	0.14±0.23	0.19±0.26	0.24±0.26
**Amygdala_R**	0.26±0.18	0.22±0.29	0.25±0.17	0.2±0.17
**Calcarine_L**	0.39±0.26	0.39±0.22	0.34±0.26	0.32±0.26
**Calcarine_R**	0.38±0.27	0.37±0.24	0.33±0.26	0.31±0.26
**Cuneus_L**	0.38±0.26	0.35±0.24	0.34±0.24	0.35±0.24
**Cuneus_R**	0.4±0.29	0.34±0.25	0.37±0.21	0.36±0.21
**Lingual_L**	0.4±0.27	0.42±0.21	0.37±0.22	0.38±0.22
**Lingual_R**	0.4±0.29	0.4±0.23	0.36±0.23	0.37±0.23
**Occipital_Sup_L**	0.4±0.27	0.42±0.28	0.44±0.19	0.46±0.19
**Occipital_Sup_R**	0.44±0.25	0.46±0.26	0.5±0.27	0.44±0.27
**Occipital_Mid_L**	0.45±0.2	0.5±0.23	0.51±0.22	0.52±0.22
**Occipital_Mid_R**	0.44±0.22	0.46±0.28	0.55±0.27	0.49±0.27
**Occipital_Inf_L**	0.44±0.24	0.52±0.2	0.49±0.22	0.48±0.22
**Occipital_Inf_R**	0.45±0.23	0.47±0.24	0.53±0.24	0.5±0.24
**Fusiform_L**	0.42±0.25	0.45±0.24	0.46±0.24	0.47±0.24
**Fusiform_R**	0.47±0.27	0.48±0.26	0.47±0.25	0.46±0.25
**Postcentral_L**	0.36±0.27	0.38±0.25	0.46±0.31	0.39±0.31
**Postcentral_R**	0.42±0.29	0.41±0.28	0.47±0.3	0.39±0.3
**Parietal_Sup_L**	0.35±0.27	0.34±0.29	0.41±0.25	0.36±0.25
**Parietal_Sup_R**	0.44±0.25	0.39±0.28	0.48±0.28	0.4±0.28

**Parietal_Inf_L**	0.2±0.23	0.19±0.32	0.26±0.25	0.2±0.25
**Parietal_Inf_R**	0.23±0.26	0.16±0.28	0.24±0.25	0.21±0.25
**SupraMarginal_L**	0.3±0.24	0.28±0.31	0.33±0.23	0.35±0.23
**SupraMarginal_R**	0.34±0.29	0.32±0.26	0.32±0.24	0.33±0.24
**Angular_L**	0.02±0.23	0.02±0.35	0.09±0.29	0.12±0.29
**Angular_R**	0.1±0.24	0.06±0.34	0.11±0.32	0.1±0.32
**Precuneus_L**	0.32±0.24	0.27±0.29	0.27±0.29	0.32±0.29
**Precuneus_R**	0.34±0.25	0.27±0.27	0.29±0.27	0.31±0.27
**Paracentral_Lobule_L**	0.39±0.21	0.44±0.21	0.39±0.21	0.43±0.21
**Paracentral_Lobule_R**	0.44±0.22	0.44±0.21	0.43±0.25	0.43±0.25
**Caudate_L**	0.17±0.23	0.21±0.25	0.25±0.21	0.26±0.21
**Caudate_R**	0.25±0.24	0.21±0.27	0.21±0.21	0.22±0.21
**Putamen_L**	0.2±0.2	0.16±0.23	0.18±0.23	0.2±0.23
**Putamen_R**	0.26±0.31	0.25±0.31	0.23±0.24	0.24±0.24
**Thalamus_L**	0.23±0.26	0.13±0.3	0.19±0.27	0.18±0.27
**Thalamus_R**	0.26±0.24	0.14±0.3	0.21±0.27	0.18±0.27
**Heschl_L**	0.37±0.23	0.44±0.22	0.35±0.27	0.32±0.27
**Heschl_R**	0.35±0.27	0.38±0.24	0.38±0.27	0.36±0.27
**Temporal_Sup_L**	0.47±0.22	0.49±0.24	0.41±0.27	0.44±0.27
**Temporal_Sup_R**	0.47±0.27	0.46±0.22	0.39±0.27	0.41±0.27
**Temporal_Pole_Sup_L**	0.36±0.19	0.32±0.27	0.29±0.31	0.33±0.31
**Temporal_Pole_Sup_R**	0.41±0.21	0.41±0.22	0.38±0.25	0.38±0.25
**Temporal_Mid_L**	0.36±0.24	0.47±0.27	0.4±0.25	0.45±0.25
**Temporal_Mid_R**	0.5±0.16	0.55±0.2	0.48±0.22	0.49±0.22
**Temporal_Pole_Mid_L**	0.24±0.18	0.25±0.22	0.27±0.27	0.28±0.27
**Temporal_Pole_Mid_R**	0.28±0.17	0.35±0.21	0.31±0.3	0.32±0.3
**Temporal_Inf_L**	0.34±0.18	0.35±0.26	0.35±0.3	0.4±0.3
**Temporal_Inf_R**	0.56±0.19	0.53±0.23	0.53±0.26	0.5±0.26
**Cerebelum_Crus1_L**	0.38±0.27	0.38±0.27	0.39±0.22	0.33±0.22
**Cerebelum_Crus1_R**	0.41±0.27	0.4±0.27	0.39±0.19	0.38±0.19
**Cerebelum_Crus2_L**	0.22±0.23	0.1±0.34	0.14±0.34	0.1±0.34
**Cerebelum_Crus2_R**	0.23±0.22	0.13±0.32	0.17±0.28	0.16±0.28
**Cerebelum_3_L**	0.26±0.18	0.24±0.24	0.29±0.2	0.22±0.2
**Cerebelum_3_R**	0.27±0.19	0.25±0.22	0.29±0.18	0.24±0.18

**Cerebelum_4_5_L**	0.38±0.21	0.44±0.22	0.38±0.22	0.4±0.22
**Cerebelum_4_5_R**	0.4±0.2	0.38±0.26	0.38±0.2	0.38±0.2
**Cerebelum_6_L**	0.44±0.27	0.49±0.2	0.44±0.17	0.41±0.17
**Cerebelum_6_R**	0.47±0.26	0.51±0.21	0.48±0.16	0.48±0.16
**Cerebelum_7b_L**	0.2±0.17	0.12±0.3	0.15±0.29	0.06±0.29
**Cerebelum_7b_R**	0.19±0.21	0.14±0.28	0.12±0.25	0.15±0.25
**Cerebelum_8_L**	0.3±0.15	0.21±0.25	0.21±0.22	0.15±0.22
**Cerebelum_8_R**	0.28±0.19	0.18±0.27	0.16±0.28	0.16±0.28
**Cerebelum_9_L**	0.18±0.22	0.11±0.24	0.13±0.22	0.09±0.22
**Cerebelum_9_R**	0.18±0.19	0.13±0.27	0.12±0.2	0.1±0.2
**Cerebelum_10_L**	0.07±0.19	0.1±0.18	0.02±0.24	0.02±0.24
**Cerebelum_10_R**	0.13±0.17	0.07±0.22	-0.05±0.2	0.02±0.2
**Vermis_3**	0.17±0.21	0.11±0.22	0.19±0.2	0.15±0.2
**Vermis_4_5**	0.31±0.27	0.28±0.25	0.28±0.22	0.3±0.22
**Vermis_6**	0.38±0.23	0.38±0.21	0.33±0.2	0.31±0.2
**Vermis_7**	0.34±0.24	0.3±0.23	0.28±0.23	0.22±0.23
**Vermis_8**	0.26±0.18	0.22±0.25	0.2±0.17	0.15±0.17
**Vermis_9**	0.12±0.22	0.13±0.24	0.09±0.16	0.06±0.16
**Vermis_10**	0.06±0.19	0.14±0.22	0.05±0.23	0.1±0.23
